# Photopic light-mediated down-regulation of local α_1A_-adrenergic signaling protects blood-retina barrier in experimental autoimmune uveoretinitis

**DOI:** 10.1038/s41598-019-38895-y

**Published:** 2019-02-20

**Authors:** Andrea Stofkova, Daisuke Kamimura, Takuto Ohki, Mitsutoshi Ota, Yasunobu Arima, Masaaki Murakami

**Affiliations:** 1 Division of Molecular Psychoimmunology, Institute for Genetic Medicine and Graduate School of Medicine, Hokkaido University, Sapporo, 060-0815 Japan; 2 Department of Physiology, Third Faculty of Medicine, Charles University, Prague, Czech Republic

## Abstract

We have reported the gateway reflex, which describes specific neural activations that regulate immune cell gateways at specific blood vessels in the central nervous system (CNS). Four types of gateway reflexes exist, all of which induce alterations in endothelial cells at specific vessels of the blood-brain barrier followed by inflammation in the CNS in the presence of CNS-autoreactive T cells. Here we report a new gateway reflex that suppresses the development of retinal inflammation by using an autoreactive T cell-mediated ocular inflammation model. Exposure to photopic light down-regulated the adrenoceptor pathway to attenuate ocular inflammation by suppressing breaching of the blood-retina barrier. Mechanistic analysis showed that exposure to photopic light down-regulates the expression of α_1A_-adrenoceptor (α_1A_AR) due to high levels of norepinephrine and epinephrine, subsequently suppressing inflammation. Surgical ablation of the superior cervical ganglion (SCG) did not negate the protective effect of photopic light, suggesting the involvement of retinal noradrenergic neurons rather than sympathetic neurons from the SCG. Blockade of α_1A_AR signaling under mesopic light recapitulated the protective effect of photopic light. Thus, targeting regional adrenoceptor signaling might represent a novel therapeutic strategy for autoimmune diseases including those that affect organs separated by barriers such as the CNS and eyes.

## Introduction

The regulation of immune responses by the nervous system represents a spectrum of inhibitory and excitatory neural pathways. Inflammatory reflexes are fundamental neural circuits mediated by the vagus nerve and are important for immune response resolution, as they prevent excessive cytokine production and tissue damage^1–5^. Gateway reflexes regulate the status of the blood-brain barrier (BBB) to establish immune cell gateways and the induction of neural inflammation^6–9^. Activation of a gateway reflex stimulates the endothelium of specific blood vessels in the central nervous system (CNS) to secrete chemokines. This secretion allows CNS-autoreactive CD4+ T cells to breach the BBB and invade the CNS, where they cause inflammation^6,8,9^. For example, sensory neural activation in the soleus muscles by gravity or electric stimulation induces chemokine expressions in the dorsal vessels of the fifth lumbar (L5) spinal cord via sympathetic nerve activation. During experimental autoimmune encephalomyelitis (EAE), an animal model of multiple sclerosis, chemokine up-regulation at the L5 vessels acts as a gateway for pathogenic CD4+ T cells specific for myelin-oligodendrocyte glycoprotein to invade the CNS from the L5 site^6^. Overall, various neural stimulations create gateways at different blood vessels in the CNS. Pain and chronic stress induce distinct immune cell gateways at the ventral vessels of the L5 cord and specific vessels beside the third ventricle, dentate gyrus, and thalamus, respectively^8,9^. Electric stimulations to muscles induce the formation of immune cell gateways at the dorsal vessels of the spinal cord where the dorsal root ganglion of the sensory neurons in the muscle is located^6^. In general, these specific neural inputs lead to the release of neurotransmitters such as norepinephrine (NE) and/or ATP at specific vessels in the BBB, which in turn enhances the expression of chemokines in the endothelium to establish gateways through which immune cells can reach the CNS^1–6,8,10,11^. In addition, we have reported that stress establishes immune cell gateways at two brain vessels sites followed by the development of microinflammation^9^. The resulting microinflammation then activates new neural pathways in a manner dependent on ATP and risks upper gastrointestinal and heart failure with sudden death. These results showed that the gateway reflex can affect the homeostasis of organs besides the brain.

To breach the BBB via a gateway reflex, the induction of massive chemokine expression by endothelial cells is critical. We identified the inflammation amplifier as the mechanism responsible. The inflammation amplifier involves co-activation of NF-κB and STAT3 in non-immune cells including endothelial cells, followed by the hyper-activation of NF-κB to express NF-κB target genes such as chemokines and IL-6^12–14^. Activation of the inflammation amplifier is critical for the development of mouse models of rheumatoid arthritis, multiple sclerosis, skin inflammation and allogeneic transplantation rejections^6,8,9,12–23^. It is known that NE and epinephrine (EPI) enhance NF-κB activation^6,24,25^, which is a molecular basis that links gateway reflexes and the inflammation amplifier^10,11^. The above examples all describe ways in which the BBB is breached. On the other hand, no mechanism dependent on specific neural activation that prevents the breaching has been identified.

A prominent feature of autoimmune posterior uveitis is chronic inflammation of the retina and choroid that commonly results in blindness. It is believed that autoreactive CD4+ T cells, particularly Th1 and Th17 cells, initiate the pathogenic process, and malfunction of the blood-retinal barrier (BRB) is considered a critical early phenomenon for the disease development^26–28^. Because retinal vessels express adrenergic receptors^29,30^ and because NE is released in the retina from sympathetic neurons from outside the eyes^31^ and retinal neurons themselves such as amacrine cells and horizontal cells produce both NE and EPI^32–35^, we hypothesized that a gateway reflex determines the BRB status by regulating NE/EPI release in the retina.

The main sensory stimulus for the retina is light, which directly affects three photoreceptor cell types in the retina: rods, cones, and melanopsin-expressing intrinsically photosensitive retinal ganglion cells (ipRGCs)^36^. Thus, different light intensities should activate different retinal neural pathways. Consistent with this theory, photopic but not scotopic light has been shown to increase the activity of retinal tyrosine hydroxylase (TH), the enzyme that synthesizes catecholamines including NE and EPI in the retina^33,37^.

In the present study, we employed two light intensities, photopic and mesopic light, and investigated BRB alterations in the initial phase of experimental autoimmune uveoretinitis (EAU) when retina-specific autoreactive CD4+ cells infiltrate the eye^38–41^. We found that exposure to photopic light significantly suppressed the inflammatory phenotype of the BRB endothelium and the recruitment of immune cells including pathogenic CD4+ T cells in the retina of EAU mice. Further, our mechanistic analysis suggests that photopic light in EAU mice down-regulates retinal α_1A_-adrenoceptor (α_1A_AR) expression, which in turn decreases chemokine and IL-6 expressions in the retina. These results suggest that photopic light can stimulate a suppressive type of gateway reflex, the light-gateway reflex, which has an anti-inflammatory effect in the retina. Our data also imply that targeting the regional adrenoceptor signal and/or retinal α_1A_AR might represent a novel preventive and/or therapeutic strategy for autoimmune diseases including those that affect organs separated by blood barriers such as the CNS and eyes.

## Results

### CD4+ T cells are the first cells to invade the eye on day 10 after immunization

**Figure 1:**
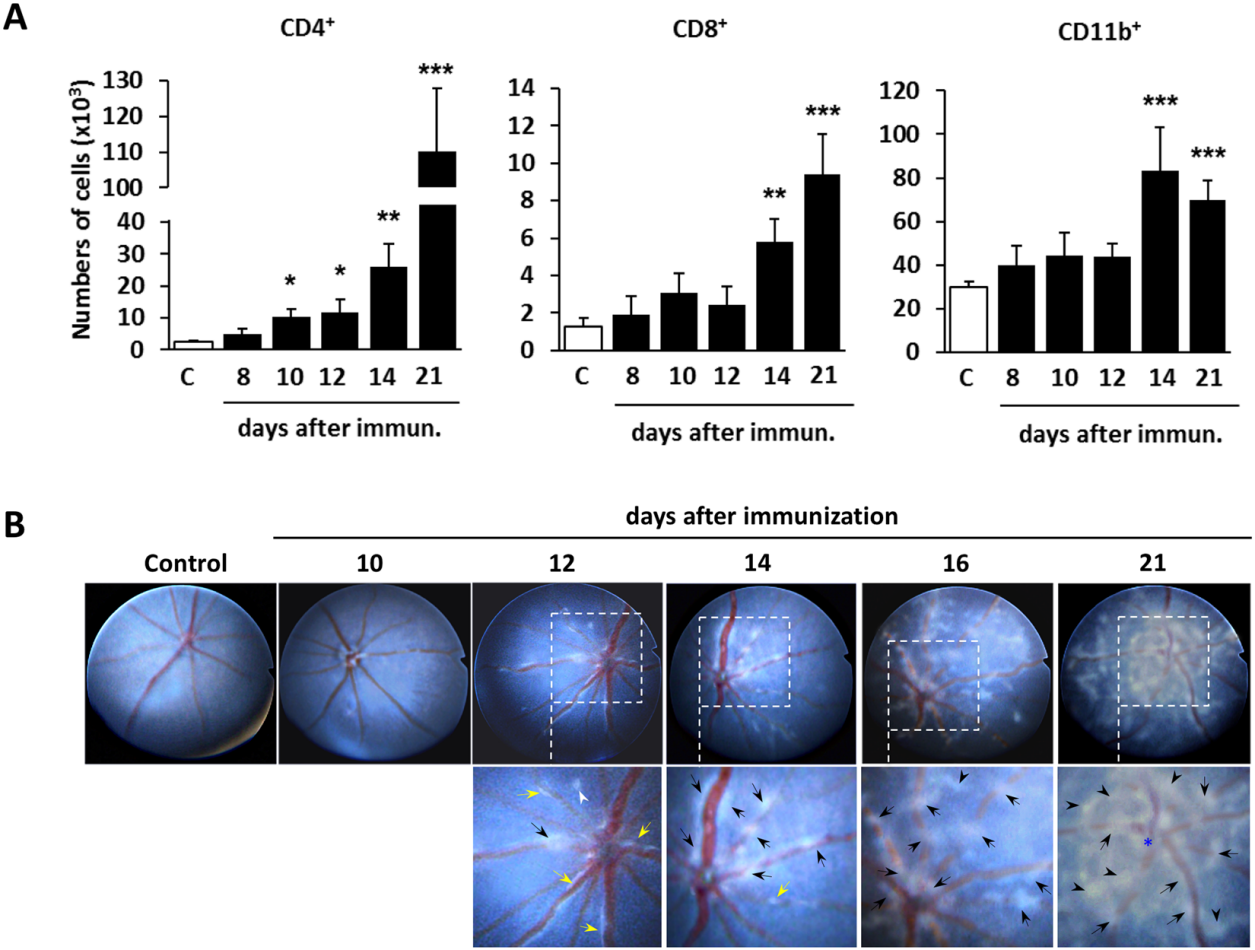
Time course of immune cell infiltration into the eye during EAU. (**A**) Time-course of leukocyte entry into the eyes quantified by flow cytometry on days 8, 10, 12, 14, and 21 after immunization. Data are expressed as means + s.e.m. (*N* = 8–10 per time point). One representative analysis of at least two independent experiments is shown. Kruskal-Wallis non-parametric ANOVA followed by Dunn’s multiple comparison test between each EAU time point and non-immunized control (C); *P < 0.05, **P < 0.01, ***P < 0.001. (**B**) Representative fundus images obtained by the TEFI system from non-immunized control and EAU mice on the indicated days after immunization. In the magnified images of the inflamed areas, the severity of retinal damage is denoted by (1) yellow arrows, retinal vasculitis with mild cuffing, (2) black arrows, retinal vasculitis with moderate to severe cuffing, (3) white arrowheads, small inflammatory infiltrates in retinal tissue, (4) black arrowheads, linear retinal lesions, and (5) blue asterisk, severe inflammation in the optic disc with papilledema.

To investigate the effect of light intensity on ocular inflammation during EAU development, we focused on the initial phase of the disease because our previous study on a multiple sclerosis model, EAE, demonstrated that neuro-immune interactions are important for triggering the breakdown of the BBB by pathogenic CD4+ T cells^6,8,9,42^. To determine the time point of the initial immune cell infiltration during the early phase of EAU, we analyzed the number of immune cells in the eyes of mice housed under 2 lux mesopic light, which is within limits of normal light intensity values for mouse cages in our animal facility, in the light phase of the experiments and 0 lux scotopic condition in the dark phase. The number of CD4+ T cells significantly increased in the eyes from day 10 after immunization, followed by an increment of other immune cells such as CD8+ T cells and CD11b+ myeloid cells on day 14 (Fig. 1A), which is consistent with the fact that EAU is a CD4+ T cell-dependent disease^38–41^. Fundoscopic analysis revealed inflammatory responses in the retinal tissue and around the retinal vessels as early as day 12 after immunization (Fig. 1B).

### Protective effect of photopic light on EAU development

**Figure 2:**
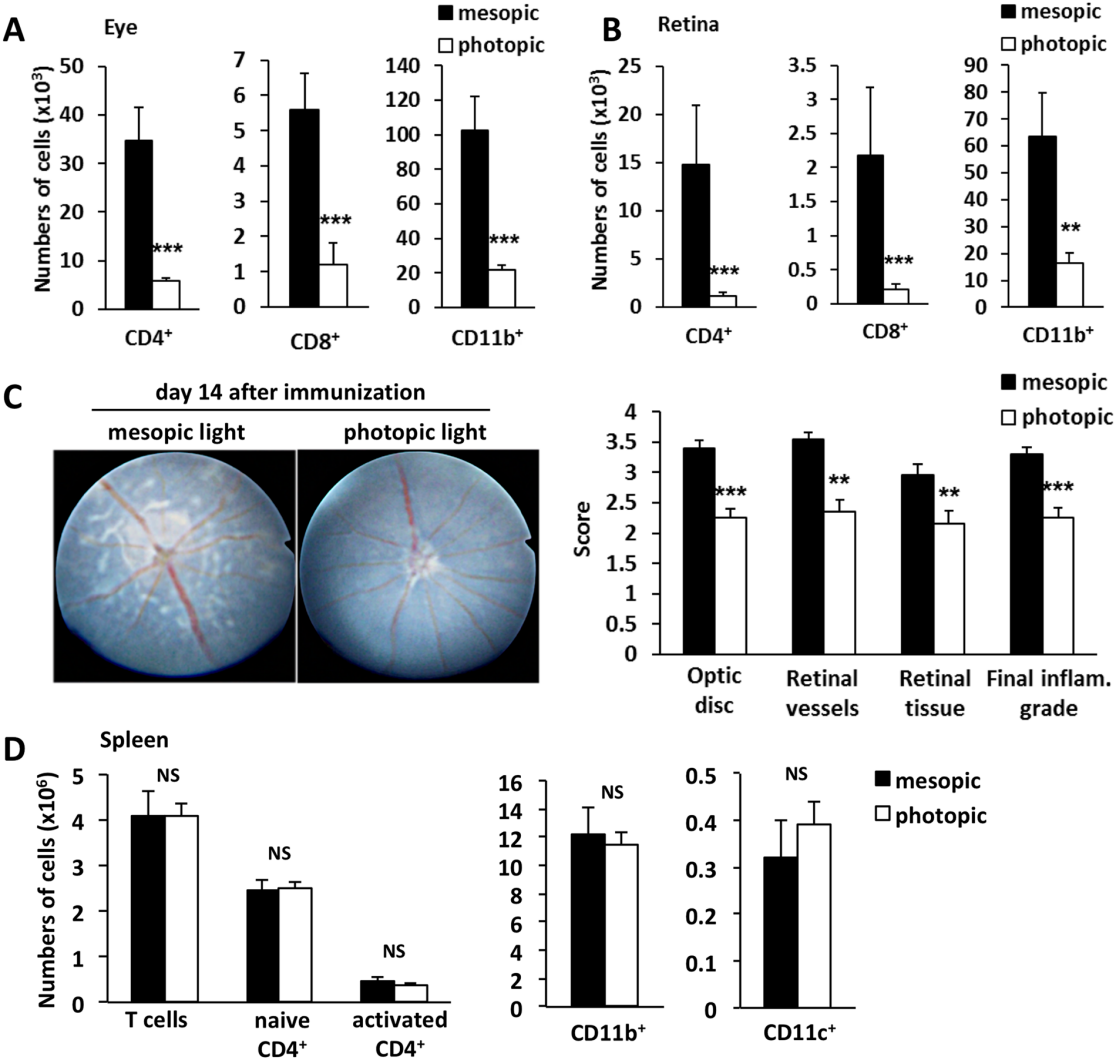
Photopic light attenuated clinical disease severity and reduced accumulation of immune cells in eye and retina. (**A**,**B**) Absolute numbers of CD4^+^, CD8^+^, and CD11b^+^ cells in eyes (A) and neural retinas (B) of EAU mice housed under mesopic or photopic light. Determined by flow cytometry on day 14 after immunization. Data are expressed as means + s.e.m. (*N* = 10 each). ***P < 0.001 by two-tailed Student’s t-test or Mann-Whitney U test. (**C**) Representative appearance of the ocular fundus recorded by the TEFI system (left) and TEFI clinical scores (right) on day 14 after immunization. Optic disc, retinal vessels and retinal tissue inflammatory changes were scored separately. The final inflammation grade represents the average of the scores of these three components. Data are expressed as means + s.e.m. (*N* = 20 each). **P < 0.01 and ***P < 0.001 by two-tailed Student’s t-test. (**D**) Absolute numbers of splenocytes determined by flow cytometry in EAU mice housed under mesopic or photopic light on day 14 after immunization. Naive CD4^+^ T cells (CD4^+^CD44^low^), activated CD4^+^ T cells (CD4^+^CD44^high^), CD11b^+^ (CD11b^high^CD11c^low^ macrophages and neutrophils) and CD11c^+^ (CD11c^high^MHC-II^high^ dendritic cells). Data are expressed as means + s.e.m. (*N* = 6 each). NS, not significant.

We next examined the role of light intensity on CD4+ T cell infiltration in the retina. We applied two light intensities during the light phase: mesopic or photopic (230 lux) from day 10 after immunization (Supplementary Fig. 1), the day when CD4+ T cells infiltrate the eyes to a significant degree (Fig. 1A). All animals were housed under 0 lux condition in the dark phase. On day 14 after immunization, mice housed under photopic light showed a significantly reduced accumulation of CD4+, CD8+ and CD11b+ cells in the eyes and neural retina (Fig. 2A,B, and Supplementary Fig. 2). Consistently, photopic light from day 10 onward decreased EAU clinical scores compared with mesopic light (Fig. 2C). However, the absolute number of splenic naïve/activated CD4+ T cells, CD11b+ cells, and CD11c+ dendritic cells were comparable in EAU mice independent of the light conditions (Fig. 2D), suggesting local retinal and not systemic immune suppression under photopic light. These results suggest that retinal inflammation is suppressed by photopic light stimulation during EAU development.

### Photopic light suppresses inflammatory phenotype of BRB endothelium and retinal chemokine expression

**Figure 3:**
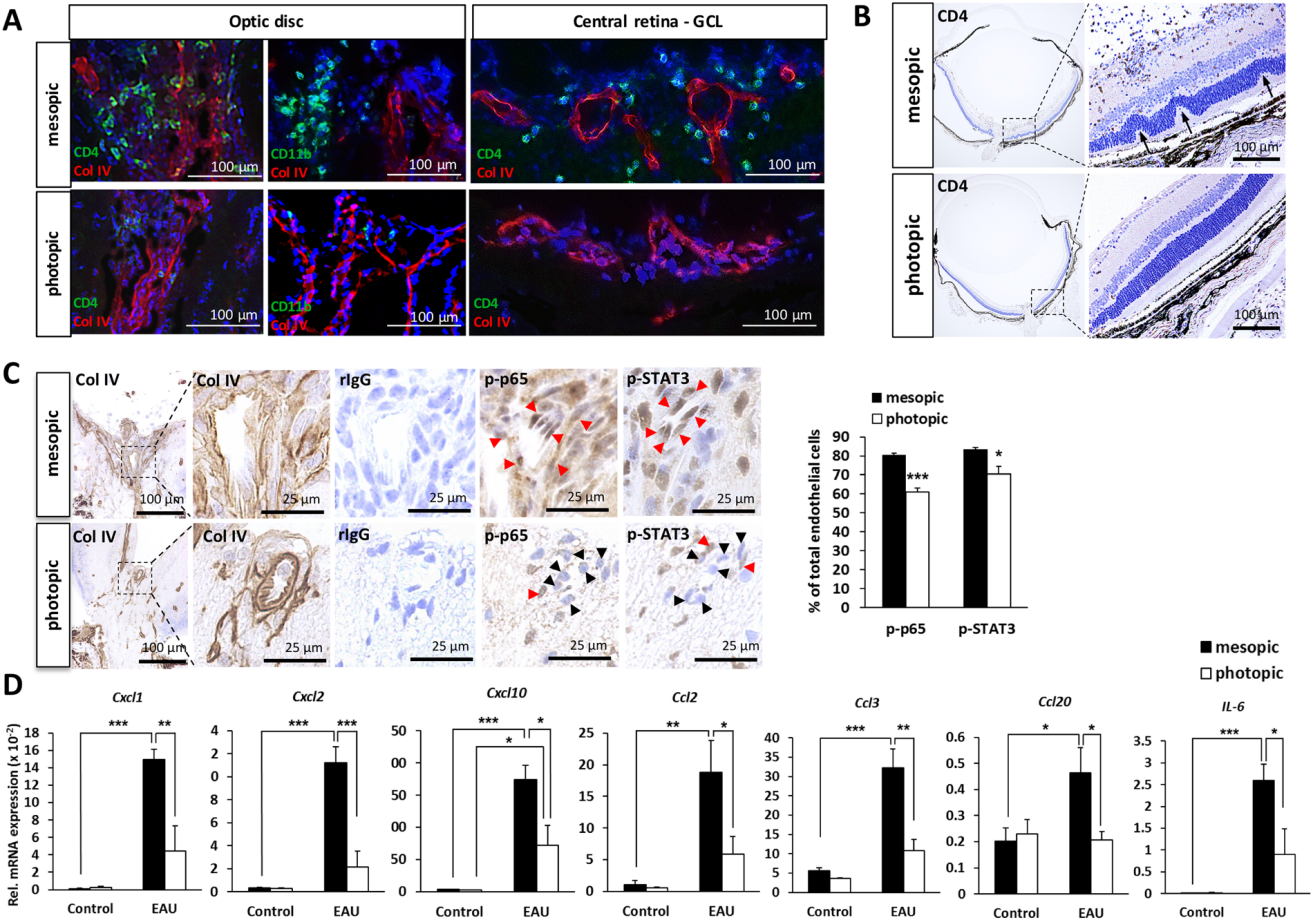
Photopic light prevented entry of immune cells in the optic disc and retina by suppressing the inflammatory status of BRB endothelium and local chemokine expression. (**A**) Inflammatory cell infiltrates around microcirculation vessels in the optic disc and central retina in EAU mice housed under mesopic or photopic light on day 14 after immunization. Green, CD4^+^ T and CD11b+ cells; red (type IV collagen; Col IV), endothelium and basal lamina; blue (Hoechst), nuclei. GCL, ganglion cell layer. (**B**) Immunohistochemical staining for the CD4^+^ cell population and retinal histopathology (arrows show retinal folds) in EAU mice housed under mesopic or photopic light. (**C**) Immunohistochemical staining for vascular marker Col IV, control rabbit IgG (rIgG), phospho-p65, and phospho-STAT3 performed on serial eye sections in EAU mice housed under mesopic or photopic light on day 14 after immunization. Red arrowheads, endothelial cells positive for p-p65 or p-STAT3 protein expression; black arrowheads, endothelial cells negative for p-p65 or p-STAT3 protein expression. The graph displays percentages of p-p65- and p-STAT3-positive vascular endothelial cells in the optic disc area in EAU mice (day 14 after immunization) exposed to mesopic or photopic light. Data are expressed as means + s.e.m. (*N* = 6 to 5 each). *P < 0.05, ***P < 0.001 by two-tailed Student’s t-test. (**D**) Chemokine and IL-6 mRNA expressions in neural retinas from intact control and EAU mice (day 14 after immunization) housed under mesopic or photopic light condition. Data are expressed as means + s.e.m. (*N* = 6 to 8 each). *P < 0.05, **P < 0.01, ***P < 0.001 by two-tailed Student’s t-test.

Because it has been reported that inflammation first develops at the optic disc in EAU mice^43^, we examined the central retinal vessels located in the middle of the optic disc in detail. On day 14 after immunization, EAU mice housed under photopic light showed very few CD4+ T and CD11b+ cells around the central retinal vessels of the optic disc (Fig. 3A). Similarly, photopic light exposure attenuated the infiltration of CD4+ T cells in the central and peripheral retina and minimized retinal structural damage including the formation of retinal folds (Fig. 3B).

We reasoned how the immune cells infiltrating the retina passed through the BRB from the blood circulation. Because we previously discovered that gateway reflexes entail a chemokine hyper-induction mechanism (i.e. the inflammation amplifier) in endothelial cells to establish immune cell gateways at specific vessels in the BBB^6,8,9,12–23^, we hypothesized that a similar mechanism is critical for breaching the BRB during EAU development. Consistently, the phosphorylation of NF-κB and STAT3, which is required for activation of the inflammation amplifier, was suppressed in the retinal endothelial cells of EAU mice housed under photopic light (Fig. 3C). Moreover, we found that the expression of chemokines and IL-6 was significantly reduced in the neural retina of EAU mice under photopic light compared to mesopic light (Fig. 3D). These results strongly suggest that photopic light diminishes chemokine and cytokine expression in the retina and protects against BRB breakdown.

### Photopic light increases eye norepinephrine and epinephrine in EAU mice

**Figure 4:**
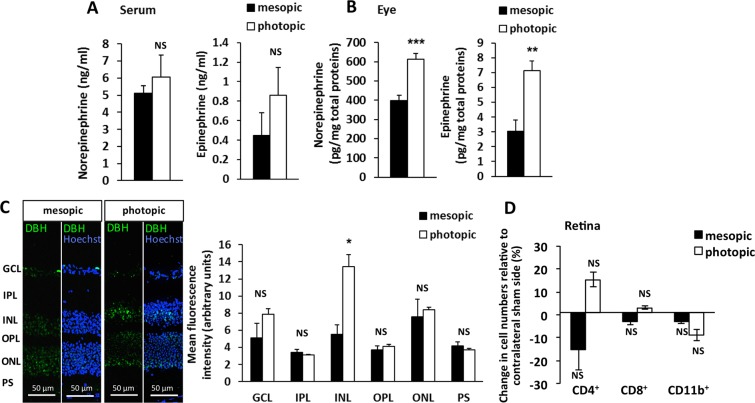
High levels of norepinephrine and epinephrine in the eye from EAU mice under photopic condition. (**A**,**B**) Norepinephrine and epinephrine levels in serum (A) and eyes (B) of EAU mice (day 14 after immunization) housed under mesopic or photopic light. Data are expressed as means + s.e.m. (*N* = 5 each). **P < 0.01, **P < 0.01 by two-tailed Student’s t-test. NS, not significant. (**C**) Immunofluorescence staining of DBH (green) protein expression and quantitative analysis of mean fluorescence intensities of DBH expression in the retinal layers of EAU mice (day 14 after immunization) housed under mesopic or photopic light. Blue, Hoechst staining of nuclei. Data are means + s.e.m. (*N* = 4 each). *P < 0.05 by two-tailed Student’s t-test. GCL, ganglion cell layer; IPL, inner plexiform layer; INL, inner nuclear layer; OPL, outer plexiform layer; ONL, outer nuclear layer; PS, photoreceptor segments. (**D**) Flow cytometric analysis of CD4^+^ T cells, CD8^+^ T cells, and CD11b^+^ cells in the neural retina (day 14 after immunization) after unilateral SCG-X in EAU mice exposed to mesopic or photopic light. The graph shows percent changes in cell numbers in the neural retina on the SCG-X side relative to the contralateral sham side in each mouse housed under mesopic or photopic conditions. Data are expressed as means + s.e.m. (*N* = 8–10 each). NS, not significant.

Given our previous observations on the importance of NE for the gateway reflexes^1–6,8–11^ and the presence of adrenergic receptors on retinal blood vessels^29,30^, we investigated whether light-mediated sensory inputs to the retina interact with the regional noradrenergic system of the eye. First, we examined changes in NE and EPI levels in the eyes and serum after photopic and mesopic light treatment. Even though we did not observe any changes in serum levels (Fig. 4A), EAU mice housed under the photopic condition exhibited more NE and EPI levels in the eyes than EAU mice housed under the mesopic condition (Fig. 4B). No significant changes in serum NE and EPI levels also suggested that the intensity of photopic light used did not induce a systemic stress response in mice. In addition, the expression level of DBH, an enzyme necessary for NE and EPI synthesis, was significantly higher in the inner nuclear layer (INL) of EAU mice housed under photopic light compared to EAU mice housed under mesopic light (Fig. 4C). Other enzymes required for NE and EPI synthesis including TH, aromatic L-amino acid decarboxylase and phenylethanolamine N-methyltransferase were also expressed in the INL as described previously (data not shown; see^33,44,45^). Although eyes are known to receive sympathetic innervation^31^, unilateral removal of the superior cervical ganglion (SCG-X) did not significantly affect immune cell accumulation in the eye compared to that on the sham-operated side (Fig. 4D and Supplementary Fig. 1). It is known that retinal neurons such as amacrine cells and horizontal cells synthesize NE and EPI, and they are localized in the INL where higher DBH expression was observed^32–35^. These results suggest that a photopic light-induced local neural pathway in the retina leads to higher NE and EPI levels in the eyes of EAU mice at least in part via resident retinal cells in the INL.

### Photopic light down-regulates α_1_AR expression in the retina

**Figure 5:**
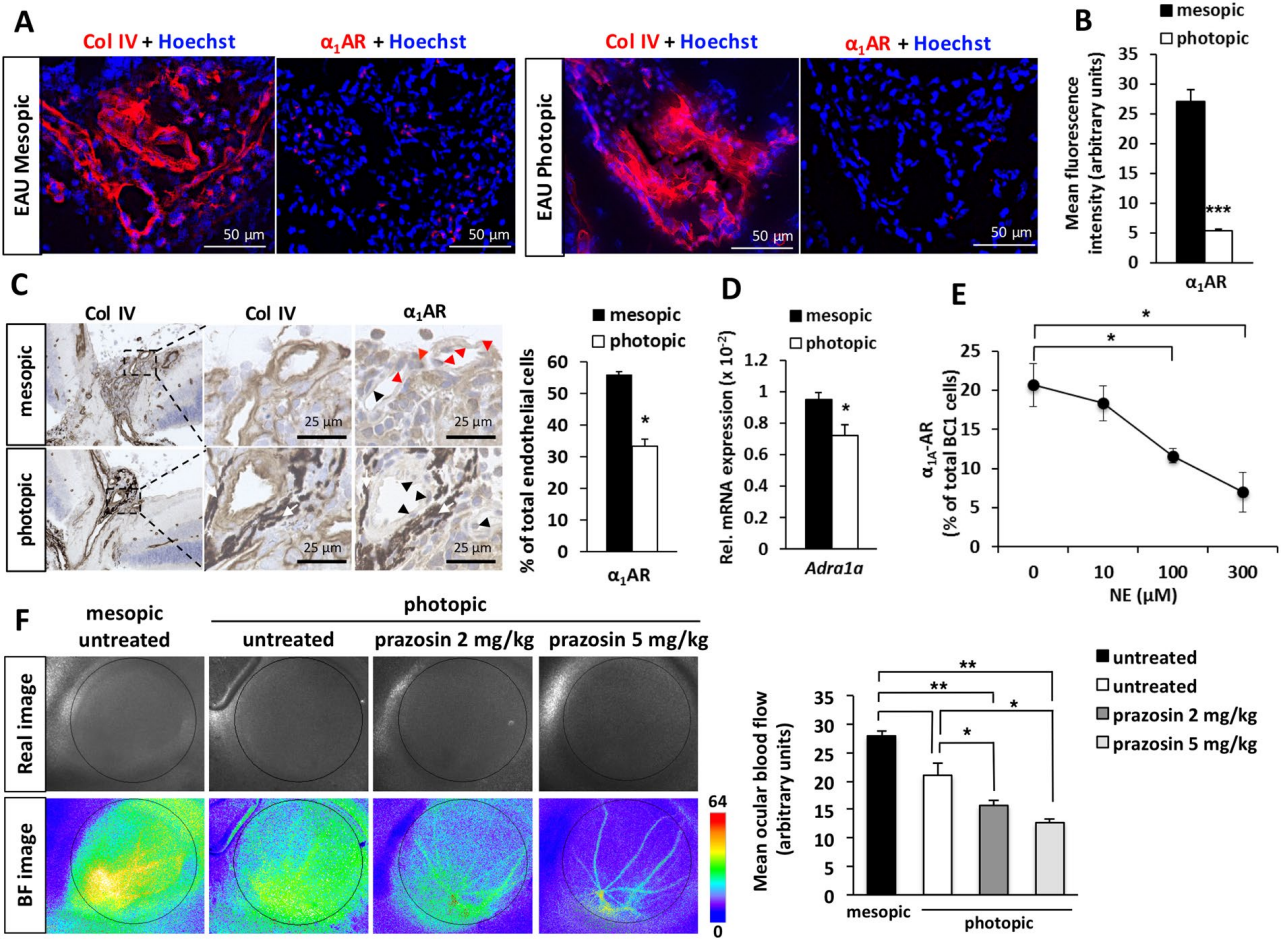
α_1A_AR expression in the retina of EAU mice exposed to mesopic or photopic light and its regulation by norepinephrine in endothelial cells. (**A**) Fluorescence microscopy images (serial sections) showing α_1_AR protein expression in vascularized areas (type IV collagen; Col IV) of the optic disc in EAU mice housed under mesopic or photopic light on day 14 after immunization. Nuclei were stained by Hoechst. (**B**) Mean fluorescence intensities of α_1_AR expressions in the optic disc vascular beds stained with Col IV in the sections shown in (**A**). Data are expressed as means + s.e.m. (*N* = 4 each). ***P < 0.001 by two-tailed Student’s t-test. (**C**) Immunohistochemistry for vascular marker Col IV, α_1_AR, and control rabbit IgG (rIgG) performed on serial eye sections of EAU mice housed under mesopic or photopic light on day 14 after immunization. Red arrowheads, endothelial cells positive for α_1_AR expression; black arrowheads, endothelial cells negative for α_1_AR expression; white arrows, eye pigment normally present in C57BL/6 mice. The graph shows percentage of α_1_AR-positive vascular endothelial cells. Data are expressed as means + s.e.m. (N = 5 each). *P < 0.05 by two-tailed Student’s t-test. (**D**) mRNA levels for Adra1a in the neural retina of EAU mice (day 14 after immunization) housed under mesopic or photopic light. Data are expressed as means + s.e.m. (*N* = 6 each). *P < 0.05 by two-tailed Student’s t-test. (**E**) Effect of 48 h NE stimulation on total protein expression of α_1A_AR in BC1 cells determined by flow cytometry. Data are expressed as means + s.e.m. (*N* = 3 each). *P < 0.05 by two-tailed Student’s t-test. (**F**) Representative real and blood flow (BF) images obtained from wild-type BALB/c mice exposed to mesopic or photopic light with or without prazosin treatment. B6 mice were not suitable for this assay due to eye pigmentation. The graph shows alterations of mean ocular blood flow determined in a region of interest (depicted by the circles in the BF images). Data are presented as means + s.e.m. (*N* = 5 each). **P < 0.01 by two-tailed Student’s t-test.

Increased NE and EPI signaling enhances NF-κB activation to promote local inflammation^6,24^. On the other hand, it is reported that NE and EPI induce ligand-dependent adrenoceptor (AR) down-regulation and desensitization^46–49^. Therefore, we next examined AR expression in the retina in order to explain reduced eye inflammation despite higher catecholamine levels. Previously, we found a reduced accumulation of pathogenic CD4+ T cells at the L5 cord in EAE mice after treatment with prazosin, a selective α_1_AR antagonist^6^ Another study also showed that blockade of α_1_AR suppressed the development of EAU in rats^50^. Therefore, we focused on the α_1_AR family. Indeed, photopic light decreased total α_1_AR protein expression in endothelial cells of the central retinal vessels in EAU mice (Fig. 5A–C), which is the site where immune cell infiltration was observed (Fig. 3A)^43^. Among the three transcripts of α_1_AR subtypes (α_1A,_ α_1B_ and α_1D_), the mRNA level of Adra1a, which encodes α_1A_AR, was decreased in EAU mice housed under photopic light (Fig. 5D). Consistently, after the stimulation of endothelial cells with NE *in vitro*, the reduction of α_1A_AR protein levels was observed (Fig. 5E). Moreover, exposure to photopic light reduced the mean ocular blood flow compared with exposure to mesopic light, which may implicate a reduction of catecholamine signaling in retinal endothelial cells (Fig. 5F). Treatment with prazosin further reduced the ocular blood flow, suggesting that α_1_AR mediates ocular blood flow control and the effect of photopic light used in this study on α_1_AR down-regulation is not saturated. These results suggest that high levels of NE and EPI under photopic light in EAU mice down-regulate α_1A_AR protein levels in the central retinal vessels, which prevented the α_1A_AR signaling required for inflammation development and immune cell accumulation.

### Blockade of α_1_AR recapitulates protective effects of photopic light in EAU mice

**Figure 6:**
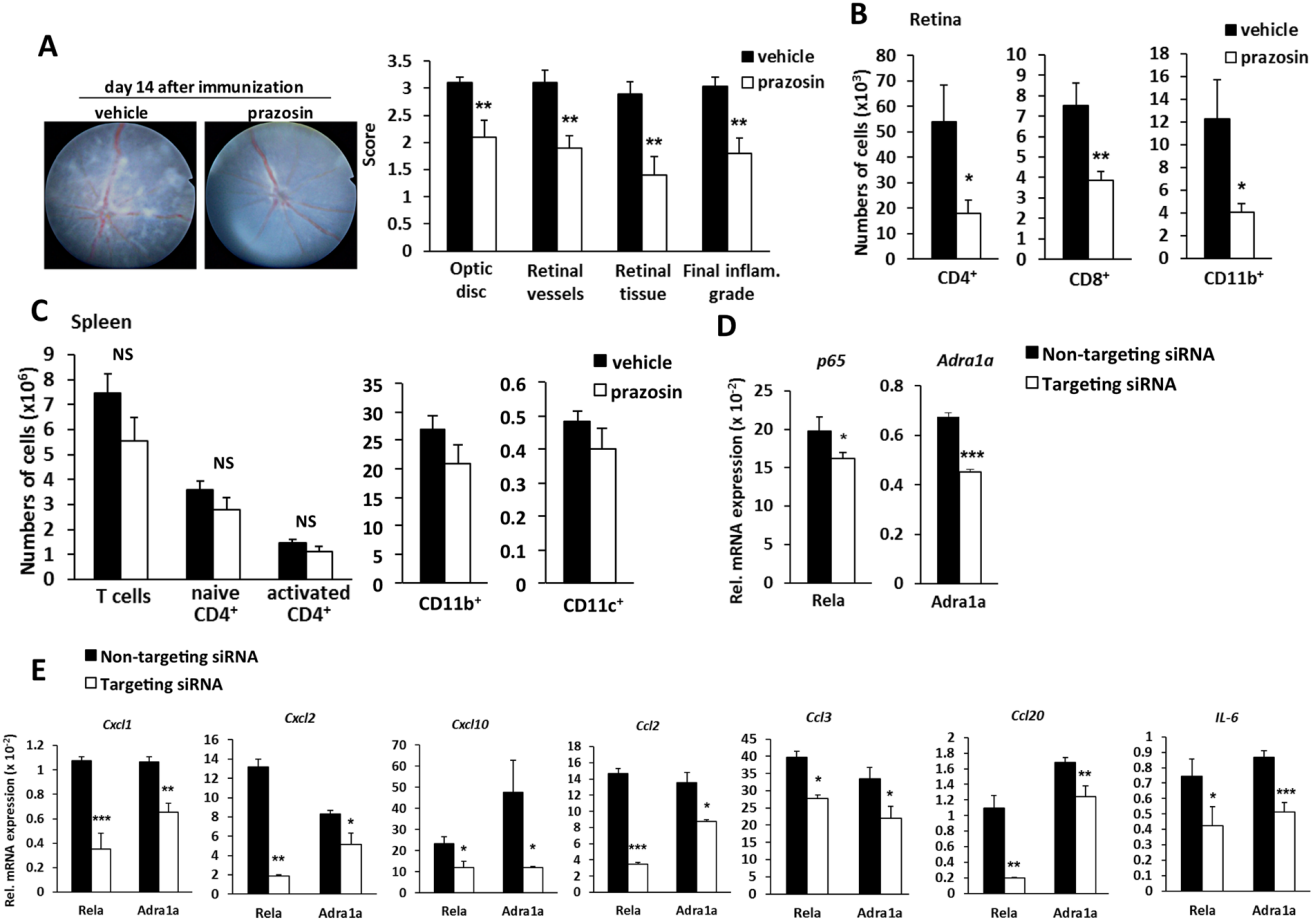
Protective effects of α_1_AR and α_1A_AR inhibition in EAU mice housed under mesopic light. (**A**) Effect of prazosin treatment on EAU clinical scores by the TEFI system on day 14 after immunization in EAU mice housed under mesopic light. Representative fundus images (left) and TEFI clinical scores (right). Optic disc, retinal vessels, and retinal tissue inflammatory changes were scored separately. The final inflammation grade represents the average of the scores of these three components. Data are presented as means + s.e.m. (*N* = 10 each). **P < 0.01 by two-tailed Student’s t-test. (**B**,**C**) Absolute numbers of CD4^+^, CD8^+^, and CD11b^+^ cells in neural retina (B) and spleen (C) determined by flow cytometry. Naive CD4^+^ T cells (CD4^+^CD44^low^), activated CD4^+^ T cells (CD4^+^CD44^high^), CD11b^+^ (CD11b^high^CD11c^low^ macrophages and neutrophils), and CD11c^+^ (CD11c^high^MHC-II^high^ dendritic cells). Data are presented as means + s.e.m. (*N* = 10 each). * *P* < 0.05 and ** *P* < 0.01 by two-tailed Student’s t-test. NS, not significant. (**D**) Knockdown efficiency of Adra1a- and Rela-targeting siRNA in the neural retina 4 days after intravitreal injection of the respective siRNAs administered on day 10 post-immunization in EAU mice housed under mesopic light. Data are expressed as means + s.e.m. (*N* = 4 each). *P < 0.05, ***P < 0.001 by two-tailed Student’s t-test. (**E**) Alterations of chemokine and IL-6 mRNA expressions in neural retinas of EAU mice housed under mesopic light 4 days after treatment with intravitreal injection of siRNA against Adra1a or Rela administered on day 10 post-immunization. Data are expressed as means + s.e.m. (*N* = 4 each). *P < 0.05, **P < 0.01, ***P < 0.001 by two-tailed Student’s t-test.

To investigate whether the down-regulation of α_1_AR-mediated signaling can explain the beneficial effect of photopic light, we used both pharmacological and genetic approaches. Prazosin treatment in EAU mice housed under mesopic light (Supplementary Fig. 1) significantly reduced clinical scores and retinal CD4+, CD8+ and CD11b+ cell infiltrates (Fig. 6A,B), observations reminiscent of the anti-inflammatory effect of photopic light. Consistently, prazosin did not influence the number of immune cells or their activation in the spleen (Fig. 6C). Moreover, a small interfering RNA (siRNA)-mediated knockdown approach (Supplementary Fig. 1) that targeted Adra1a successfully knocked down Adra1a transcript (Fig. 6D) and significantly suppressed chemokine and IL-6 expression in the retina of EAU mice housed under mesopic light (Fig. 6E). We also examined siRNA-mediated knockdown of Rela (NF-κB p65), which is an essential transcription factor for the inflammation amplifier. As expected, intravitreal injection of Rela-targeting siRNA also significantly reduced retinal chemokine and IL-6 mRNA levels (Fig. 6D,E). All these data suggest that α_1_AR signaling and NF-κB activation in the retina have a pivotal role on EAU development and that the anti-inflammatory effect of photopic light during EAU development is at least in part mediated by α_1_AR down-regulation.

## Discussion

We have previously demonstrated using a mouse model of multiple sclerosis, EAE, that gateway reflexes induce NE-mediated alterations of endothelial cells at specific blood vessels in the CNS to regulate the BBB status. To date, four types of gateway reflexes have been demonstrated, and all cause CNS inflammation in the presence of CNS-autoreactive immune cells in blood^6,8,9,11^. In the present study, we identified a suppressive type of gateway reflex by using a retinal inflammation model and showed that photopic light-mediated neural activation suppresses breaching of the BRB even in the presence of organ-specific T cells.

We examined the CD44 and CD62L levels of T cells that infiltrated the retina on day 14 after immunization (early phase). Over 90% of these T cells had the CD44^high^ CD62L^low^ activated phenotype, as expected (Supplementary Fig. 3). It is reported that splenocytes from unimmunized mice that have been adoptively transferred to IRBP-immunized mice around the peak of the disease migrate to the retina^51^, suggesting that bystander cells including antigen-nonspecific naïve and activated T cell populations infiltrate the organ once the blood-retinal barrier is fully breached. The current study was designed to address the effect of light intensity on the initial entry phase of immune cells to the retina. It is possible that both naïve and activated T cells could be found at a later time point close to the peak of the disease, potentially affecting the disease development.

**Figure 7:**
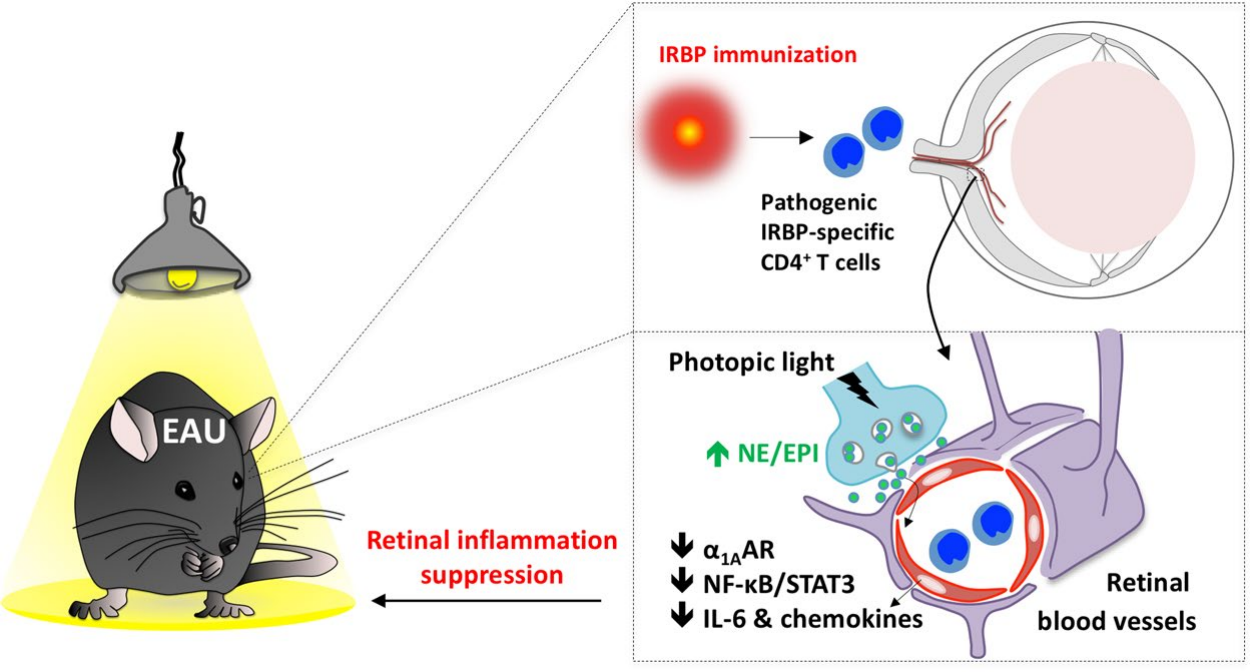
A summary diagram of suppression of retinal inflammation by photopic light. Exposure of EAU mice to photopic light causes down-regulation of retinal α_1A_AR expression by high epinephrine (EPI) and norepinephrine (NE) release from retinal neurons, which significantly inhibits activation of NF-κB and STAT3 and decreases chemokine and IL-6 expressions in the retina, leading to a reduced recruitment of immune cells including IRBP-specific pathogenic CD4+ T cells.

Our results suggest that photopic light treatment inhibits the accumulation of immune cells in the retina during EAU development by down-regulating α_1A_AR signaling in endothelial cells of the BRB. This conclusion is supported by four observations. First, exposure to photopic light from the onset of the pre-clinical phase of EAU resulted in a significant attenuation of immune cell infiltrates in the eyes. Second, photopic light in EAU mice suppressed the inflammatory phenotype of the BRB endothelium including the activation of NF-κB and STAT3 and the retinal gene expression of various chemokines and cytokines that attract and/or activate immune cells. Third, NE and EPI levels in the eyes were higher after photopic light treatment than under mesopic light treatment in EAU mice, which correlates with DBH expression in INL neurons and is associated with the down-regulation of retinal α_1A_AR expression. Finally, blockade of α_1_AR or retinal α_1A_AR knockdown in EAU mice housed under mesopic light showed the protective effects on EAU development seen in photopic light treatment. These results strongly suggest that the light-mediated α_1_AR-adrenergic pathway in the retina is involved in disruption of the BRB during EAU development (Fig. 7). This is in contrast to our previous studies showing that at the induction and relapse phases of the transfer EAE model, NE enhances NF-κB activity in the dorsal and ventral vessels of the fifth lumbar spinal cord, respectively, to promote disease progression^6,8^. However, the transfer EAE model shows a transiently increasing clinical score followed by recovery from the symptoms^8^. Accordingly, there is some negative regulation of inflammation during EAE development *in vivo*. We hypothesize that down-regulation of α_1_AR contributes to this negative regulation during the remission of EAE.

There are at least two potential sources of NE and EPI in the eye: sympathetic neurons^31^ and retinal neurons such as amacrine and horizontal cells^32–35^. We found that eliminating sympathetic innervation of the eye by SCG-X had a minimal effect on EAU development. Because the number of accumulated immune cells in the retina of EAU mice on the SCG-X side did not significantly differ from that on the sham-operated side in either mesopic or photopic light condition, we suggest only a minor contribution by sympathetic neurons to NE and EPI content in the eye. However, EAU mice under photopic light showed high expressions of DBH enzyme in the INL, indicating that INL cells are at least in part a local producer of NE and EPI during EAU development under photopic condition. Consistent with this observation, it is known that retinal neurons located in the INL increase their activity in response to photopic light stimulation and that exposure to photopic light increases retinal EPI levels in both normal and SCG-X rats^33,52^. Thus, we hypothesize that resident retinal cells in the INL including amacrine and horizontal cells, rather than SCG sympathetic neurons, are responsible for the photopic light-induced NE and EPI production in the eye and that this production is linked to EAU amelioration. Moreover, amacrine and horizontal cells form neurovascular units with capillaries in retinal vascular plexuses and are essential for maintaining homeostasis of the intraretinal vasculature^53^, suggesting their ability to regulate the phenotype and remodeling of BRB endothelial cells.

Light detected in the retina activates distinct local neural retinal circuits depending on light intensity via photoreceptors. Photopic light intensity activates directly cone- as well as ipRGC-mediated retinal pathways, while single-photon scotopic light in darkness activates rod- and ipRGC-mediated retinal pathways^36,54^. On the other hand, all three photoreceptor cell types can respond to mesopic light^36,55,56^. Therefore, under photopic light condition, neural pathways originating most likely from cones and/or ipRGCs contribute to the DBH expression in noradrenergic amacrine and horizontal cells. Although it is known that amacrine and horizontal cells communicate directly with ipRGCs and cones, respectively, or with both photoreceptor systems through bipolar cells^57^, the precise pathway responsible for the activation of noradrenergic neurons in the INL in response to photopic light remains elusive. Our findings here, which show the biological importance of these photopic light-activated noradrenergic retinal neurons as immune regulators to maintain retinal homeostasis, may accelerate the discovery of specific neural pathways that convert photopic light into a vascular effect that protects BRB integrity.

An earlier pharmacologic study in a rat EAU model demonstrated the amelioration of EAU after systemic treatment with the α_1_AR blocker prazosin^50^. We confirmed this finding in EAU mice housed under mesopic condition, observing that prazosin treatment from day 10 post-immunization suppressed the local retinal but not systemic immune response in a manner similar to that found under photopic light condition. In addition, we observed that photopic light exposure reduced ocular blood flow. It is reported that after EAU induction, shear stress in retinal veins is reduced, and this reduction is negatively correlated with the rolling and sticking effectiveness of leukocytes^51^. On the other hand, it is also known that fluid shear stress promotes lymphocyte migration across endothelial barriers in cytokine-activated human umbilical vascular endothelial cells^58^. Since it is also suggested that retinal veins have higher shear stress than normal fenestrated endothelium^26,51^, we hypothesize that the photopic light decreased ocular blood flow to a level that inefficiently promotes transendothelial migration.

We also investigated the effect of other adrenergic antagonists on EAU development, including the nonselective βAR blocker propranolol (20 mg/kg; daily i.p.), the β_1_AR blocker atenolol (25 mg/kg; daily i.p.), and the nonselective αAR blocker phentolamine (5 mg/kg; daily i.p.), but we did not observe any significant effects of these drugs on EAU outcome (data not shown), suggesting that α_1_AR plays a major role in inflammation development during EAU. Further, we showed the down-regulation of α_1A_AR expression after treatment with NE *in vitro*. Consistent with this finding, it is reported that the expressions of many G protein-coupled receptors, including αARs, are down-regulated after long-term exposure to an agonist^49,59,60^. Therefore, we hypothesize that NE down-regulates α_1_AR molecules on the cell surface due to the internalization of NE-α_1_AR complexes and subsequent transcriptional regulation of α_1_AR. Consistent with this notion, it was also reported that NE treatment reduces receptor expression on the cell surface^49,59,60^. Thus, we suggest that the higher level of NE in the eyes of EAU mice housed under photopic condition suppressed retinal α_1A_AR expression compared to EAU mice housed under mesopic condition, which resulted in a decrease of α_1_AR signaling and NF-κB activation^6,24^.

The IRBP_1-20_-induced EAU in B6 mice that we used here is associated with chronic inflammation involving mainly the posterior segment of the eye, thereby serving as a good model of human posterior uveitis^61^. According to reports from clinical studies, the majority of patients with uveitis are not photophobic. In one study, the percentage of cases associated with photophobia was 3.4% in pediatric uveitis^62^ and 5.8% among autoimmune uveitis in both children and adult patients^27^. In another study, photophobia was the presenting symptom in 1.8%, 3.2% and 6.8% cases of panuveitis, posterior uveitis and anterior uveitis, respectively^63^, allowing the use of light stimulation for a potential treatment. Indeed, there are several papers that report the effectiveness of light treatment on patients suffering from eye inflammatory diseases^64–67^. However, the sensitivity to light can be different between mice and humans^68^, and thus optimal light intensity or timing to produce the anti-inflammatory effect in humans has yet to be determined. In our study, a five-day exposure to photopic light of 230 lux starting from the preclinical stage of EAU significantly ameliorated retinal inflammation. Since the photopic light treatment used in this study modulated endothelial cells of the retinal vasculature to suppress the expression of proinflammatory factors including chemokines, we assume that photopic light treatment is effective particularly at the early stage of the disease rather than the late phase, when the BRB is completely breached. It is known that bright light of 5,000 lux activates a nociceptive pathway in trigeminal nerves^69^, which might affect the disease status of EAU by pain-induced responses including the pain-gateway reflex^8^. Whether various intensities or durations of photopic light exposure differently influence the outcome of EAU is an important point to address in the future.

In summary, we demonstrated a previously unknown suppressive role of light function in the regulation of immune cell accumulation across the BRB, which also underscores the role of the retinal α_1A_-noradrenergic pathway in local inflammation development in EAU mice (Fig. 7). This effect is reminiscent of gateway reflexes, in which specific regional neural activations establish immune cell gateways by changing the status of endothelial cells of the BBB in the CNS. We previously reported gravity-, electric-, pain- and stress-gateway reflexes^6,8,9,11^. Here we propose that photopic light induces another gateway reflex, the light-gateway reflex. In contrast to previously discovered gateway reflexes, the light-gateway reflex provides the first evidence that gateway reflexes can have a suppressive effect on local inflammation. Overall, the light-gateway reflex, which is induced by an enhanced regional adrenoceptor signal followed by the down-regulation of retinal α_1A_AR, might represent a novel preventive and/or therapeutic strategy for autoimmune diseases, particularly those in which blood-tissue barrier breakdown is a key factor for the disease development.

## Materials and Methods

### Animals

Female wild type C57BL/6 (B6) and BALB/c mice 6 weeks old with specific-pathogen free (SPF) status were purchased from SLC (Tokyo, Japan) and housed in an SPF animal facility at the Institute for Genetic Medicine (Hokkaido University, Sapporo, Japan). All mice were adapted to a 13 h/11 h light/dark photoperiod in the animal facility for seven days before experiments and had at libitum access to food and water. Experiments were performed in agreement with the guidelines of the Institutional Animal Care and Use Committees of Hokkaido University. Experimental protocols were approved by the Institutional Animal Care and Use Committees of Hokkaido University (No. 14-0083).

### Induction of EAU and treatment with prazosin

EAU was induced in B6 mice by active immunization with 100 μL emulsion containing 200 µg human interphotoreceptor retinoid-binding protein (IRBP_1–20_) (amino acids 1–20 (GPTHLFQPSLVLDMAKVLLD); custom product by Sigma-Aldrich, Tokyo, Japan) and 1 mg Mycobacterium tuberculosis strain H37Ra (Becton, Dickinson and Company, Franklin Lakes, NJ, USA) in complete Freund’s adjuvant (CFA). The IRBP_1–20_/CFA emulsion was injected subcutaneously into the base of the tail (day 0), and mice were injected intravenously with 200 μL (0.2 μg) of Bordetella pertussis toxin (Sigma-Aldrich) dissolved in saline on days 0 and 2. All mice were housed under illumination of 2 lux from day 0 to day 10 post-immunization. Afterwards, mice were divided into two groups and were held either in mesopic (2 lux) or photopic (230 lux) condition until day 14 post-immunization. The light intensity was measured with a digital lux meter (LX-1000; Custom Japan Co., Osaka). The light level during the dark phase of the day/night cycle was 0 lux in all experiments. Treatment with prazosin was started from day 10 post-immunization in mice subjected to mesopic (2 lux) condition from day 0 until the end of the experiment (day 14). Prazosin was dissolved in 5% glucose solution acidified with acetic acid to 0.25% and administered intraperitoneally every 12 h for 5 days at a dose of 5 mg/kg body weight. Control mice received the vehicle solution.

### Imaging of the eye fundus

Clinical manifestation of EAU was scored using topical endoscopy fundus imaging (TEFI), a technique adapted from Paques *et al*.^70^ with the following modifications. An endoscope was replaced with a 180 mm long borescope (MK Modular Mini-Scope, MK017-009-000-62; Olympus, Tokyo, Japan) with a 1.7 mm outer diameter, a direction of view of 0°, a field of view of 62° and a depth of field of 1 mm - ∞. The borescope was connected to an Olympus PEN digital camera (E-P5 with a 4/3-inch Live MOS image sensor and 16.05 million camera effective pixels) through adapters (OM Adapter MF-2 and AK-1M/SM-R; Olympus) without placing an additional magnifying lens between the borescope and the camera. A LED light source (ILD-3; Olympus) attached to the borescope through a flexible fiber optic cable was used to provide illumination of the fundus. The images were transferred to a computer, and the contrast and brightness were adjusted in Preview (Apple, Cupertino, CA, USA). Fundus images were scored according to the TEFI clinical grading system described previously^43^.

For the fundoscopy procedure, mice were anesthetized with 2% isoflurane in an oxygen-enriched air mixture (with 0.4 L/min oxygen flow rate) inhaled by a mask, and pupils were dilated with topical tropicamide 0.4% (Sandol MY; Nitten Nippon Tenganyaku Kenkyujo, Nagoya, Japan).

### Measurement of ocular blood flow

Ocular blood flow with particular emphasis on the retinal circulation was measured by a laser speckle flowmetry according to a published method^71^ with modification. In brief, laser light was delivered to the retina through an optical fiber positioned on the eye in a manner to obtain signals predominantly from the retinal vessels, and signals of the choroidal vasculature were imaged minimally.

Because of the strong signal reduction due to tissue pigment in the eyes of B6 mice, albino mice (BALB/c) were used in all ocular blood flow experiments. For retina imaging, 780 nm laser light was outputted from the OmegaFlo-Lab LDF-C1 laser unit (Omegawave, Tokyo, Japan) and irradiated on the eye though a custom-made 140 μm optical fiber (Omegawave) inserted into a 25-gauge needle, as described previously^71^. The ocular blood flow was imaged using a two-dimensional Laser Blood Flow Imager Omegazone OZ-1, and the data were analyzed with Laser Image Analyzer software (Omegawave).

Ocular blood flow was recorded under mesopic (2 lux) or photopic (230 lux) light after 1 h adaptation of the mice to the respective light condition. The effect of prazosin on ocular blood flow was investigated 1.5 h or 2.5 h after prazosin intraperitoneal injection.

### Flow cytometry

Mouse eyes were enucleated following transcardial perfusion with PBS. Eyes or retinas were minced and dispersed in 1 mg/mL collagenase D (Roche, Basel, Switzerland) in RPMI medium (10% FCS) for 1 h at 37 °C. Spleens were mechanically dissociated in RPMI medium. Cell surface antigens of eye or retinal cell suspensions were stained in the presence of 2.4G2 antibody and the following anti-mouse antibodies by BioLegend (San Diego, CA, USA): anti-CD11c (clone N418), anti-CD45.2 (clone 104), anti-CD3 (clone 145-2C11), anti-CD4 (clone RM4-5) and anti-CD8 (clone 53-6.7) conjugated with FITC, APC, Pacific Blue, PerCP, and PE-Cy7, respectively; and by eBioscience (San Diego, CA, USA): anti-CD19 (clone MB19-1) and anti-CD11b (clone M1/70) conjugated with FITC and PE, respectively. FITC-labeled anti-CD44 (clone IM7), PE-labeled anti-CD62L (clone MEL-14), APC-labeled CD90.2 (clone 53-2.1), APC-Cy7-labeled anti-CD45 (clone 30-F11) and Pacific blue anti-CD8 (clone 53-6.7) from BioLegend were also used. In order to analyze splenocyte populations, cells were stained in the presence of 2.4G2 antibody using the following anti-mouse antibodies: Pacific Blue-conjugated anti-CD3 (clone 145-2C11; BioLegend), PE-Cy7-conjugated anti-CD4 (clone RM4-5; eBioscience), FITC-conjugated anti-CD44 (clone IM7; eBioscience), APC-conjugated anti-CD11c (clone N418; BioLegend), APC-conjugated anti-MHC class II (clone M5/114.15.2; BioLegend), APC-conjugated anti-CD11c (clone N418; Biolegend), FITC-conjugated anti-CD11b (clone M1/70; Biolegend), Pacific Blue-conjugated anti-MHC class II (clone AF6-120.1; Biolegend), and PE-conjugated anti-CD45R/B220 (clone RA3-6B2; BD Biosciences Pharmingen, San Diego, CA, USA).

BC-1 cell suspension was incubated with anti-α_1A_AR rabbit monoclonal antibody (EPR9691(B); dilution 1:1000; Abcam) plus 2.4G2 antibody (dilution 1:200) in FACS buffer on ice for 1 h. The secondary antibody used was Alexa Fluor 488 donkey anti-rabbit IgG (H + L) (dilution 1:2000; Life Technologies) on ice for 1 h. Primary antibody unlabeled samples and positive controls (HepG2 cells) were used under the same conditions. In order to obtain a total protein expression of α_1A_AR, simultaneous fixation and permeabilization of the cells using BD Cytofix/Cytoperm™ Fixation/Permeablization Kit (BD Biosciences, San Jose, CA) were performed prior to staining with antibodies.

Flow cytometry data were acquired using CyAn Advanced Digital Processing (ADP) High-Performance Flow Cytometer (DakoCytomation, Tokyo, Japan) and analyzed by FlowJo software (Tree Star, Ashland, OR, USA).

### RNA isolation and quantitative real-time PCR

On day 14 post-immunization, mice were sacrificed under mesopic (2 lux) or photopic (230 lux) light by overdose of pentobarbital and transcardially perfused with 0.01 M PBS. Collected retinas without retinal pigment epithelium were immediately processed for total RNA extraction using AllPrep DNA/RNA Mini Kit (Qiagen, Hilden, Germany). RT-qPCR was done using KAPA SYBR FAST ABI Prism qPCR Kit or KAPA PROBE FAST ABI prism qPCR kit (Kapa Biosystems, Wilmington, MA, USA) and the specific mouse primers and probes listed in Tables 1 and 2. The relative mRNA expression of each gene was calculated using the standard curve method. All genes were normalized to the endogenous control gene *Hprt*.

**Table 1:** Sequences of primers and probes used for real-time qPCR.

Gene name	Symbol	Primers (forward) 5′-3′	Primers (reverse) 5′-3′	TaqMan probe 5′-3′
Adrenergic receptor, alpha 1a	Adra1a	GCTTCTTTCTGAAAATGCTTCTGAA	GCCACCGAGAGGATCACTAAA	FAM-CCCGCCAGCACAGGTGAACATTTCTAAGG-TAMRA
C-C motif chemokine ligand 2	Ccl2	TGTCCCAAAGAAGCTGTAGTTTTTG	GGTTCCGATCCAGGTTTTTAATGTA	FAM-CCTTCTTGGGGTCAGCACAGACCTCTCT-TAMRA
C-C motif chemokine ligand 3	Ccl3	TCCACGCCAATTCATCGTTGA	CGGTTTCTCTTAGTCAGGAAAATGA	FAM-CCAGCAGCCTTTGCTCCCAGCCAG-TAMRA
C-C motif chemokine ligand 20	Ccl20	AGGAAGAAAAGAAAATCTGTGTGC	TCTTCTTGACTCTTAGGCTGAGG	FAM-AGCCCTTTTCACCCAGTTCTGCTTTGGA-TAMRA
Chemokine (C-X-C motif) ligand 1	Cxcl1	CCACACTCAAGAATGGTCGC	CGTTACTTGGGGACACCTTTTAGC	FAM-TGCCTTGACCCTGAAGCTCCCTTGGTT-TAMRA
Chemokine (C-X-C motif) ligand 2	Cxcl2	GTCATAGCCACTCTCAAGGGC	GTCAGTTAGCCTTGCCTTTGTTC	FAM-AAAAGTTTGCCTTGACCCTGAAGCCCC-TAMRA
Chemokine (C-X-C motif) ligand 10	Cxcl10	ACGTGTTGAGATCATTGCCAC	GGCTAAACGCTTTCATTAAATTCTTG	FAM-TCCGGATTCAGACATCTCTGCTCATCATTC-TAMRA
Hypoxanthine guanine phosphoribosyl transferase	Hprt	AGCCCCAAAATGGTTAAGGTTG	CAAGGGCATATCCAACAACAAAC	FAM-ATCCAACAAAGTCTGGCCTGTATCCAACAC-TAMRA
Interleukin 6	IL-6	AGGAGACTTCACAGAGGATACCA	GCAAGTGCATCATCGTTGTTCA	FAM-CCTGTCTATACCACTTCACAAGTCGGAGGC-TAMRA

**Table 2:** Sequences of primers used for SYBR Green real-time qPCR.

Gene name	Symbol	Primers (forward) 5′-3′	Primers (reverse) 5′-3′
Hypoxanthine guanine phosphoribosyl transferase	Hprt	GATTAGCGATGATGAACCAGGTT	CCTCCCATCTCCTTCATGACA
RELA Proto-Oncogene, NF-Kappa-B p65 subunit	Rela (p65)	GAGTTCCAGTACTTGCCAGACAC	TGAAAGGACTCTTCTTCATGATACTC

### Immunohistochemistry

After transcardial perfusion, eyes were collected and frozen or paraffin blocks were made. The frozen blocks were cut into 10 μm sections using an adhesive film (Cryofilm type IIIC (UF16); SECTION-LAB, Hiroshima, Japan) and a cryostat microtome (Leica CM3050; Leica Microsystems) as described previously^6,8,72^. Tissue sections were dehydrated in 100% ethanol, fixed in 4% paraformaldehyde for 15 min and transferred into PBS. The sections were blocked with 2% BSA in PBS (sections stained for cell surface markers) or Tris-buffered saline/0.1% Tween 20 (sections stained for phospho-c-Fos) for 30 min at room temperature and then incubated overnight at 4 °C with the following anti-mouse primary antibodies (diluted in 2% BSA in PBS): rabbit anti-collagen IV (dilution 1:400; Abcam, Cambridge, UK), rabbit anti-α_1_AR (dilution 1:100; Abcam), rabbit anti-dopamine beta hydroxylase (dilution 1:100; Abcam), biotinylated rat anti-CD4 (dilution 1:200; clone RM4-5; BioLegend), and biotinylated rat anti-CD11b (dilution 1:100; clone M1/70; BioLegend). In the case of using biotinylated antibody, the sections were additionally treated with Avidin/Biotin blocking kit (Vector Laboratories, Burlingame, CA, USA) before incubation with primary antibody. Subsequently, the sections were washed twice in PBS and incubated with Hoechst 33342 fluorescent stain (at 1:10000 dilution in PBS; Life Technologies) and secondary antibodies, Alexa Fluor 647 goat anti-rabbit IgG (H + L) or Alexa Fluor 546 streptavidin conjugate (at 1:500 dilution in PBS; Life Technologies) for 1 h at room temperature.

Paraffin blocks were sectioned to 5-μm thickness. After deparaffinization of the sections, antigen retrieval was performed in 10 mM sodium citrate buffer (pH 6) or 10 mM Tris/1 mM EDTA buffer (pH 8.5). Endogenous peroxidase activity was blocked by 3% H_2_O_2_ solution for 10 min, followed by incubation of the sections in blocking solution (VECTASTAIN Elite ABC kit, Vector Laboratories) for 1 h at room temperature. Next, sections were incubated overnight at 4 °C with the following rabbit primary antibodies: anti-CD4 (dilution 1:200; clone RM4-5; BioLegend), anti-phospho-(Tyr705)-STAT3 (dilution 1:200, Cell Signaling Technology), anti-phospho-(Ser276)-p65 (dilution 1:500, Sigma-Aldrich), anti-collagen IV (dilution 1:500; Abcam), and anti-α_1_AR (dilution 1:100; Abcam). Rabbit IgG (dilution 1:200; Cell Signaling Technology) was used as isotype control. VECTASTAIN Elite ABC kit (Vector Laboratories) and the manufacturer’s protocol were applied for incubation with secondary antibody and streptavidin-HRP reagent. Signals were visualized using DAB substrate (ImmPACT DAB, Vector Laboratories). Hematoxylin (Wako chemicals, Richmond, VA, USA) was used for nuclear counter-staining.

Sections were examined under a BZ-9000 BioRevo fluorescence microscope (Keyence, Osaka, Japan). The acquired microscope images were analyzed using ImageJ software (NIH, Bethesda, MD, USA), and the image contrast and brightness were adjusted using BZ II Analyzer software (Keyence).

### Cell culture and stimulation experiments

A type 1 collagen positive endothelial cell line (BC-1)^73^ was kindly provided by Dr. M. Miyasaka (Osaka University). BC-1 cells were seeded into 24-well plates at 50,000 cells/well and treated with L-(−)-norepinephrine-(+)-bitartrate (10, 100 and 300 μM; EMD Millipore, Billerica, MA) in Dulbecco’s Modified Eagle Medium (supplemented with 10% FBS; Gibco by Life Technologies) for 48 h after 2 h serum starvation. Cells were then dissociated with 0.05% Trypsin/0.53 mM EDTA (Nacalai Tesque, Kyoto, Japan), pelleted and suspended in RP10 medium, and immediately used for flow cytometry analysis.

### Measurement of catecholamine concentrations

Mice were injected with pentobarbital under mesopic or photopic condition on day 14 post-immunization between 8–11 a.m. Blood was collected by cardiac puncture in order to obtain serum samples. Afterwards, mice were perfused with 0.01 M PBS, and the eyes were enucleated, snap-frozen on dry ice, and stored at −80 °C until homogenates were prepared. Eyes were homogenized in RIPA lysis buffer (1 mL per 100 mg of tissue; Cell Signaling Technology) containing Protease Inhibitor Cocktail, Phosphatase Inhibitor Cocktail 2 and 3 (1 mL per 100 mL of lysis buffer; Sigma-Aldrich) and sodium metabisulfite (Sigma-Aldrich) at a final concentration of 4 mM to prevent the degradation of catecholamine. The homogenization was performed using a Polytron PT1600E homogenizer (Kinematica, Luzern, Switzerland) at a speed of 30,000 rpm for 1 min. Additionally, eye homogenates were sonicated in a Biodisruptor sonication device (Diagenode, Denville, NJ) for 5 min (30 s on/off per minute cycle) at 4 °C.

The lysates were centrifuged at 15,000 × g for 10 min at 4 °C, and the supernatant was used to determine catecholamine concentrations. Commercially available enzyme-linked immunosorbent assay (ELISA), 2-CAT (A-N) Research ELISA kit (#BA E-5400; LDN, Nordhorn, Germany) was used for the quantification of NE and EPI levels in serum and eye lysates following the manufacturer’s instructions. The limit of sensitivity for serum and eye lysate NE was 20 pg/mL and 10 pg/mL, respectively, and for serum and eye EPI it was 50 pg/mL and 25 pg/mL, respectively. The eye lysate catecholamine concentrations were normalized to the milligram of tissue protein content, as determined using a Bradford protein assay (Bio-Rad, Hercules, CA).

### Intravitreal injection of siRNA

On day 10 after immunization, mice were anesthetized with 2% isoflurane in an oxygen-enriched air mixture (with 0.4 L/min oxygen flow rate) inhaled by mask and kept under anesthesia during the procedure. Mice received an intravitreal injection of Accell SMARTpool Adra1a or Accell SMARTpool Rela/p65 into the right eye and non-targeting Accell siRNA (GE Dharmacon, Lafayette, CO) into the left eye at a dose of 1 μL (100 μM siRNA dissolved in sterile, RNase free 0.01 M PBS) per eye using a graduated pulled glass pipette. A 30-gauge needle was used to poke a hole in the sclera surface at the level of pars plana. A pipette was carefully inserted into the vitreous space through the poked hole, and the siRNA solution was delivered. To avoid fluid reflux, the pipette was kept in place for 20 s and then gently withdrawn. All mice that received an intravitreal injection of siRNA were housed under mesopic light from day 0 to day 14 after immunization.

### Superior cervical ganglionectomy (SCG-X)

Female B6 mice underwent unilateral removal of SCG under isoflurane anesthesia using a 2.5% oxygen mixture. The surgery was performed under a dissection microscope. The animal was secured in supine position on a heating pad, the ventral neck region was shaved and disinfected, and a middle vertical incision was made. The carotid artery was identified and displaced laterally. The SCG was exposed behind the carotid bifurcation and excised by transecting the internal and external carotid nerves and the cervical sympathetic preganglionic nerves as described previously^69^. In the contralateral, sham-operated side, the SCG was exposed without excision. The successful removal of the SCG was confirmed by blepharoptosis on the ganglionectomized side. EAU was induced 4 days after surgery, and mice were housed under mesopic condition only or mesopic condition followed by photopic condition from day 10 post-immunization until being sacrificed on day 14 post-immunization.

### Statistical analyses

Statistical analyses were performed using Statistica software (StatSoft Inc, Tulsa, OK) and GraphPad software (GraphPad.com). According to the distribution of the data and number of groups, Student’s t-test, Mann-Whitney U test or ANOVA tests were used as indicated in the figure legends. P values less than 0.05 were considered statistically significant.

## Supplementary information

FigS1-S3

## References

[1] Tracey KJ (2012). Immune cells exploit a neural circuit to enter the CNS. Cell.

[2] Andersson U, Tracey KJ (2012). Neural reflexes in inflammation and immunity. The Journal of experimental medicine.

[3] Deutschman CS, Tracey KJ (2014). Sepsis: current dogma and new perspectives. Immunity.

[4] Tracey KJ (2016). Reflexes in Immunity. Cell.

[5] Pavlov VA, Tracey KJ (2017). Neural regulation of immunity: molecular mechanisms and clinical translation. Nature neuroscience.

[6] Arima, Y. *et al*. Regional neural activation defines a gateway for autoreactive T cells to cross the blood-brain barrier. *Cell* **148**, 447–457, https://doi.org/S0092-8674(12)00088-8 (2012). 10.1016/j.cell.2012.01.022 22304915

[7] Andersson U, Tracey KJ (2012). Reflex principles of immunological homeostasis. Annu Rev Immunol.

[8] Arima Y (2015). A pain-mediated neural signal induces relapse in murine autoimmune encephalomyelitis, a multiple sclerosis model. eLife.

[9] Arima Y (2017). Brain micro-inflammation at specific vessels dysregulates organ-homeostasis via the activation of a new neural circuit. eLife.

[10] Sabharwal L (2014). The Gateway Reflex, which is mediated by the inflammation amplifier, directs pathogenic immune cells into the CNS. J Biochem..

[11] Ohki T, Kamimura D, Arima Y, Murakami M (2017). Gateway reflex, a new paradigm of neuro-immune interaction. Clin Exp Neuroimmunol..

[12] Ogura H (2008). Interleukin-17 Promotes Autoimmunity by Triggering a Positive-Feedback Loop via Interleukin-6 Induction. Immunity..

[13] Atsumi T (2014). Inflammation amplifier, a new paradigm in cancer biology. Cancer research.

[14] Nakagawa I, Kamimura D, Atsumi T, Arima Y, Murakami M (2015). Role of Inflammation Amplifier-Induced Growth Factor Expression in the Development of Inflammatory Diseases. Critical reviews in immunology.

[15] Murakami, M. *et al*. Local microbleeding facilitates IL-6- and IL-17-dependent arthritis in the absence of tissue antigen recognition by activated T cells. *The Journal of experimental medicine* **208**, 103–114, https://doi.org/jem.20100900 (2011). 10.1084/jem.20100900 PMC3023133 21220456

[16] Murakami M (2013). Disease-association analysis of an inflammation-related feedback loop. Cell reports.

[17] Lee J (2013). IL-6 amplifier activation in epithelial regions of bronchi after allogeneic lung transplantation. Int Immunol.

[18] Harada M (2015). Temporal expression of growth factors triggered by epiregulin regulates inflammation development. Journal of immunology.

[19] Meng J (2016). Breakpoint Cluster Region-Mediated Inflammation Is Dependent on Casein Kinase II. Journal of immunology.

[20] Atsumi T (2017). Rbm10 regulates inflammation development via alternative splicing of Dnmt3b. Int Immunol.

[21] Okuyama Y (2018). Bmi1 Regulates IkappaBalpha Degradation via Association with the SCF Complex. Journal of immunology.

[22] Tanaka Y (2018). Presenilin 1 Regulates NF-kappaB Activation via Association with Breakpoint Cluster Region and Casein Kinase II. Journal of immunology.

[23] Fujita, M. *et al*. NEDD4 Is Involved in Inflammation Development during Keloid Formation. *The Journal of investigative dermatology*, 10.1016/j.jid.2018.07.044 (2018). 10.1016/j.jid.2018.07.044 30273597

[24] Bierhaus A (2003). A mechanism converting psychosocial stress into mononuclear cell activation. Proc Natl Acad Sci USA.

[25] Perez DM, Papay RS, Shi T (2009). alpha1-Adrenergic receptor stimulates interleukin-6 expression and secretion through both mRNA stability and transcriptional regulation: involvement of p38 mitogen-activated protein kinase and nuclear factor-kappaB. Mol Pharmacol.

[26] Crane IJ, Liversidge J (2008). Mechanisms of leukocyte migration across the blood-retina barrier. Semin Immunopathol.

[27] Prete M (2014). Autoimmune uveitis: a retrospective analysis of 104 patients from a tertiary reference center. J Ophthalmic Inflamm Infect.

[28] Horai R, Caspi RR (2011). Cytokines in autoimmune uveitis. J Interferon Cytokine Res.

[29] Haselton FR, Dworska EJ, Hoffman LH (1998). Glucose-induced increase in paracellular permeability and disruption of beta-receptor signaling in retinal endothelium. Invest Ophthalmol Vis Sci.

[30] Bohmer T (2014). The alpha(1)B -adrenoceptor subtype mediates adrenergic vasoconstriction in mouse retinal arterioles with damaged endothelium. Br J Pharmacol.

[31] McDougal DH, Gamlin PD (2015). Autonomic control of the eye. Compr Physiol.

[32] Park DH, Joh TH, Anwar M, Ruggiero DA (1988). Biochemical evidence for presence of dopamine beta-hydroxylase in rat retina. Brain Res.

[33] Hadjiconstantinou M, Cohen J, Neff NH (1983). Epinephrine: a potential neurotransmitter in retina. J Neurochem.

[34] Ishimoto I (1989). Co-localization of adrenergic receptors and vitamin-D-dependent calcium-binding protein (calbindin) in the dopaminergic amacrine cells of the rat retina. Neurosci Res.

[35] Chen Z, Jia W, Kaufman PL, Cynader M (1999). Immunohistochemical localization of dopamine-beta-hydroxylase in human and monkey eyes. Curr Eye Res.

[36] Yau KW, Hardie RC (2009). Phototransduction motifs and variations. Cell.

[37] Witkovsky P (2004). Activity-dependent phosphorylation of tyrosine hydroxylase in dopaminergic neurons of the rat retina. J Neurosci.

[38] Avichezer D, Silver PB, Chan CC, Wiggert B, Caspi RR (2000). Identification of a new epitope of human IRBP that induces autoimmune uveoretinitis in mice of the H-2b haplotype. Invest Ophthalmol Vis Sci..

[39] Peng Y (2007). Characterization of IL-17+ interphotoreceptor retinoid-binding protein-specific T cells in experimental autoimmune uveitis. Invest Ophthalmol Vis Sci.

[40] Liu X, Lee YS, Yu CR, Egwuagu CE (2008). Loss of STAT3 in CD4+ T cells prevents development of experimental autoimmune diseases. Journal of immunology.

[41] Hohki S (2010). Blockade of interleukin-6 signaling suppresses experimental autoimmune uveoretinitis by the inhibition of inflammatory Th17 responses. Exp Eye Res.

[42] Mori Y (2014). Early pathological alterations of lower lumbar cords detected by ultrahigh-field MRI in a mouse multiple sclerosis model. Int Immunol.

[43] Xu H (2008). A clinical grading system for retinal inflammation in the chronic model of experimental autoimmune uveoretinitis using digital fundus images. Exp Eye Res.

[44] Baetge EE, Behringer RR, Messing A, Brinster RL, Palmiter RD (1988). Transgenic mice express the human phenylethanolamine N-methyltransferase gene in adrenal medulla and retina. Proc Natl Acad Sci USA.

[45] Kralj-Hans I, Tibber M, Jeffery G, Mobbs P (2006). Differential effect of dopamine on mitosis in early postnatal albino and pigmented rat retinae. J Neurobiol.

[46] Rokosh DG (1996). Alpha1-adrenergic receptor subtype mRNAs are differentially regulated by alpha1-adrenergic and other hypertrophic stimuli in cardiac myocytes in culture and *in vivo*. Repression of alpha1B and alpha1D but induction of alpha1C. The Journal of biological chemistry.

[47] Bengtsson T, Cannon B, Nedergaard J (2000). Differential adrenergic regulation of the gene expression of the beta-adrenoceptor subtypes beta1, beta2 and beta3 in brown adipocytes. The Biochemical journal.

[48] Lei B, Zhang Y, Han C (2001). Sustained norepinephrine stimulation induces different regulation of expression in three alpha1-adrenoceptor subtypes. Life Sci.

[49] Akinaga J (2013). Differential phosphorylation, desensitization, and internalization of alpha1A-adrenoceptors activated by norepinephrine and oxymetazoline. Mol Pharmacol.

[50] Ruiz-Moreno JM, Misiuk-Hojlo M, Thillaye B, de Kozak Y (1992). Suppression of experimental autoimmune uveoretinitis by prazosin, an alpha 1-adrenergic receptor antagonist. Curr Eye Res.

[51] Xu H (2004). Reduction in shear stress, activation of the endothelium, and leukocyte priming are all required for leukocyte passage across the blood–retina barrier. Journal of leukocyte biology.

[52] Yoshida K, Kawamura K, Imaki J (1993). Differential expression of c-fos mRNA in rat retinal cells: regulation by light/dark cycle. Neuron.

[53] Usui Y (2015). Neurovascular crosstalk between interneurons and capillaries is required for vision. J Clin Invest.

[54] Do MT (2009). Photon capture and signalling by melanopsin retinal ganglion cells. Nature.

[55] Asteriti S, Gargini C, Cangiano L (2014). Mouse rods signal through gap junctions with cones. eLife.

[56] Zele AJ, Cao D (2014). Vision under mesopic and scotopic illumination. Front Psychol.

[57] Masland RH (2012). The neuronal organization of the retina. Neuron.

[58] Cinamon G, Shinder V, Alon R (2001). Shear forces promote lymphocyte migration across vascular endothelium bearing apical chemokines. Nature immunology.

[59] Heck DA, Bylund DB (1997). Mechanism of down-regulation of alpha-2 adrenergic receptor subtypes. J Pharmacol Exp Ther.

[60] Leeb-Lundberg LM, Cotecchia S, DeBlasi A, Caron MG, Lefkowitz RJ (1987). Regulation of adrenergic receptor function by phosphorylation. I. Agonist-promoted desensitization and phosphorylation of alpha 1-adrenergic receptors coupled to inositol phospholipid metabolism in DDT1 MF-2 smooth muscle cells. The Journal of biological chemistry.

[61] Singh VK, Biswas S, Anand R, Agarwal SS (1998). Experimental autoimmune uveitis as animal model for human posterior uveitis. Indian J Med Res.

[62] Smith JA (2009). Epidemiology and course of disease in childhood uveitis. Ophthalmology.

[63] Chung H, Choi DG (1989). Clinical analysis of uveitis. Korean J Ophthalmol.

[64] Lavoie MP (2009). Evidence of a biological effect of light therapy on the retina of patients with seasonal affective disorder. Biol Psychiatry.

[65] Geneva II (2016). Photobiomodulation for the treatment of retinal diseases: a review. Int J Ophthalmol.

[66] Merry GF, Munk MR, Dotson RS, Walker MG, Devenyi RG (2017). Photobiomodulation reduces drusen volume and improves visual acuity and contrast sensitivity in dry age-related macular degeneration. Acta Ophthalmol.

[67] Beirne K, Rozanowska M, Votruba M (2017). Photostimulation of mitochondria as a treatment for retinal neurodegeneration. Mitochondrion.

[68] Lucas RJ (2014). Measuring and using light in the melanopsin age. Trends in neurosciences.

[69] Okamoto K, Tashiro A, Chang Z, Bereiter DA (2010). Bright light activates a trigeminal nociceptive pathway. Pain.

[70] Paques M (2007). Panretinal, high-resolution color photography of the mouse fundus. Invest Ophthalmol Vis Sci.

[71] Srienc, A. I., Kurth-Nelson, Z. L. & Newman, E. A. Imaging retinal blood flow with laser speckle flowmetry. *Front Neuroenergetics* **2**, 10.3389/fnene.2010.00128 (2010). 10.3389/fnene.2010.00128 PMC2950742 20941368

[72] Kawamoto T (2003). Use of a new adhesive film for the preparation of multi-purpose fresh-frozen sections from hard tissues, whole-animals, insects and plants. Arch Histol Cytol.

[73] Zhang Y (1998). Production of interleukin-11 in bone-derived endothelial cells and its role in the formation of osteolytic bone metastasis. Oncogene..

