# Supplementary material for: Photopic light-mediated down-regulation of local α1A-adrenergic signaling protects blood-retina barrier in experimental autoimmune uveoretinitis
Source: Sci Rep. 2019 Feb 20;9:2353. doi: 10.1038/s41598-019-38895-y (PMC6382936; doi:10.1038/s41598-019-38895-y)
Supplement: Supplementary file 1 — FigS1-S3 [file 41598_2019_38895_MOESM1_ESM.pdf]

Supplementary information

Photopic light-mediated down-regulation of local α_{1A}-adrenergic signaling protects blood-retina barrier in experimental autoimmune uveoretinitis

[1,2] Andrea Stofkova, [1]Daisuke Kamimura, [1]Takuto Ohki, [1]Mitsutoshi Ota, [1]Yasunobu Arima, and [1]Masaaki Murakami

[1]Division of Molecular Psychoimmunology, Institute for Genetic Medicine and Graduate School of Medicine, Hokkaido University, Sapporo 060-0815, Japan
[2]Department of Physiology, Third Faculty of Medicine, Charles University, Prague, Czech Republic

Fig. S1

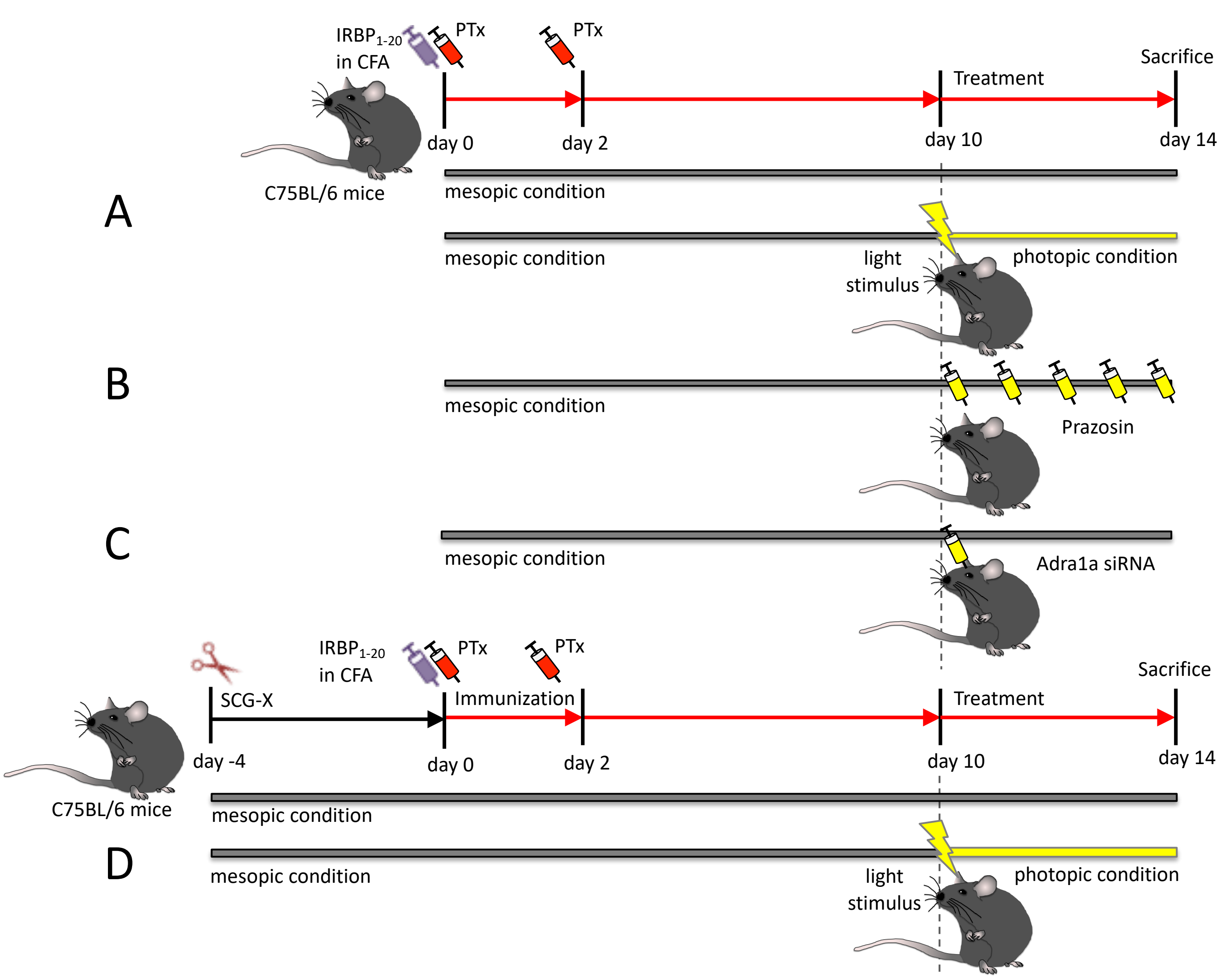

Supplementary Figure S1. Experimental design

Schematic illustration of experimental protocols for Figs. 2, 3, 4, 5A-5C (A), Figs. 6A-6C (B), Figs. 6D-6E (C), and Fig. 4D (D). CFA, complete Freund's adjuvant; IRBP, interphotoreceptor retinoid-binding protein; PTx, pertussis toxin; SCG-X, Superior cervical ganglionectomy.

Fig. S2

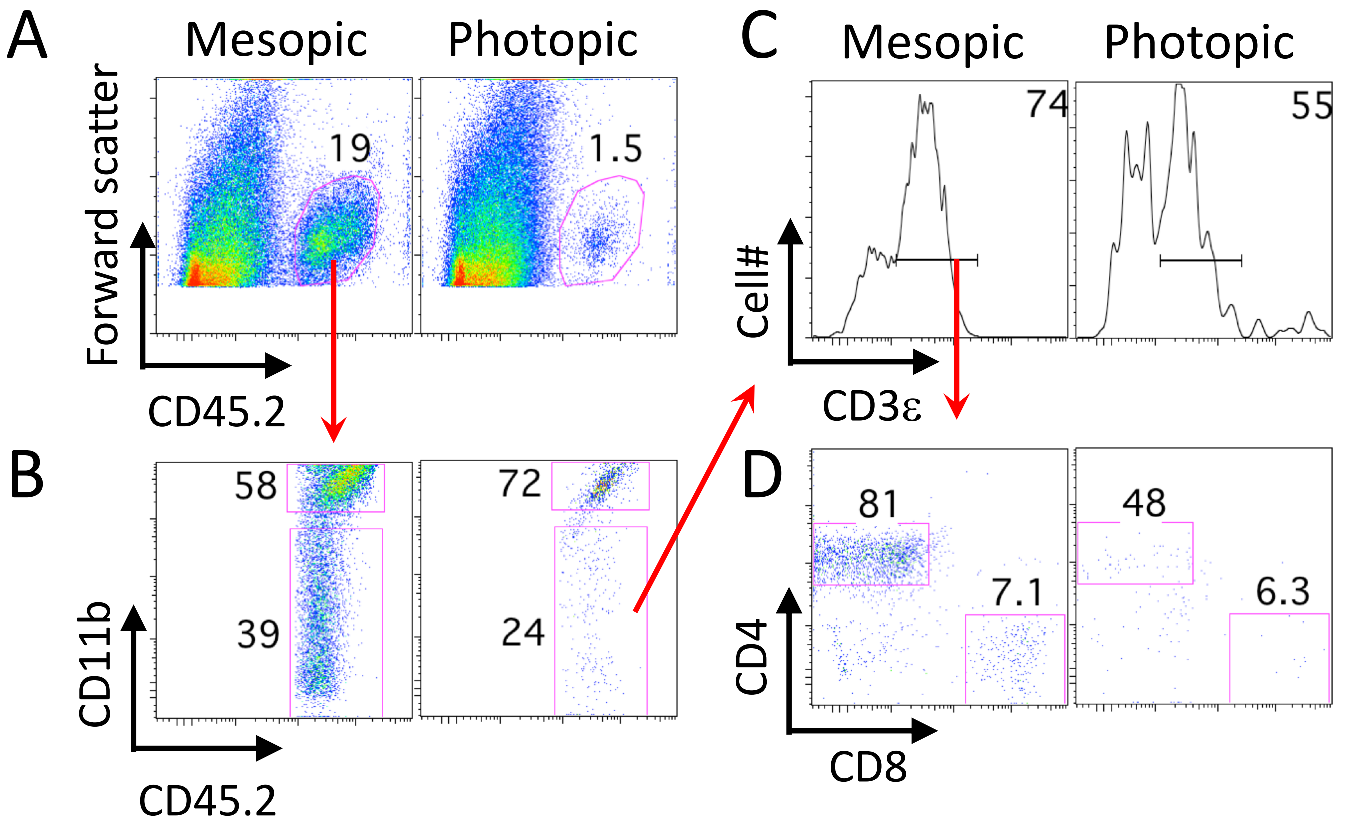

Supplementary Figure S2. Representative FACS plots of retinal samples

Collagenase D-treated retinal cells were stained with fluorescent-conjugated antibodies for FACS. CD45.2+ immune cells (A) were gated on CD11b+ or CD11b- fractions (B). CD11b- fractions then were separated by CD3e expressions to gate T cell populations (C). In (D), CD4+ and CD8+ T cells were gated.

Fig. S3

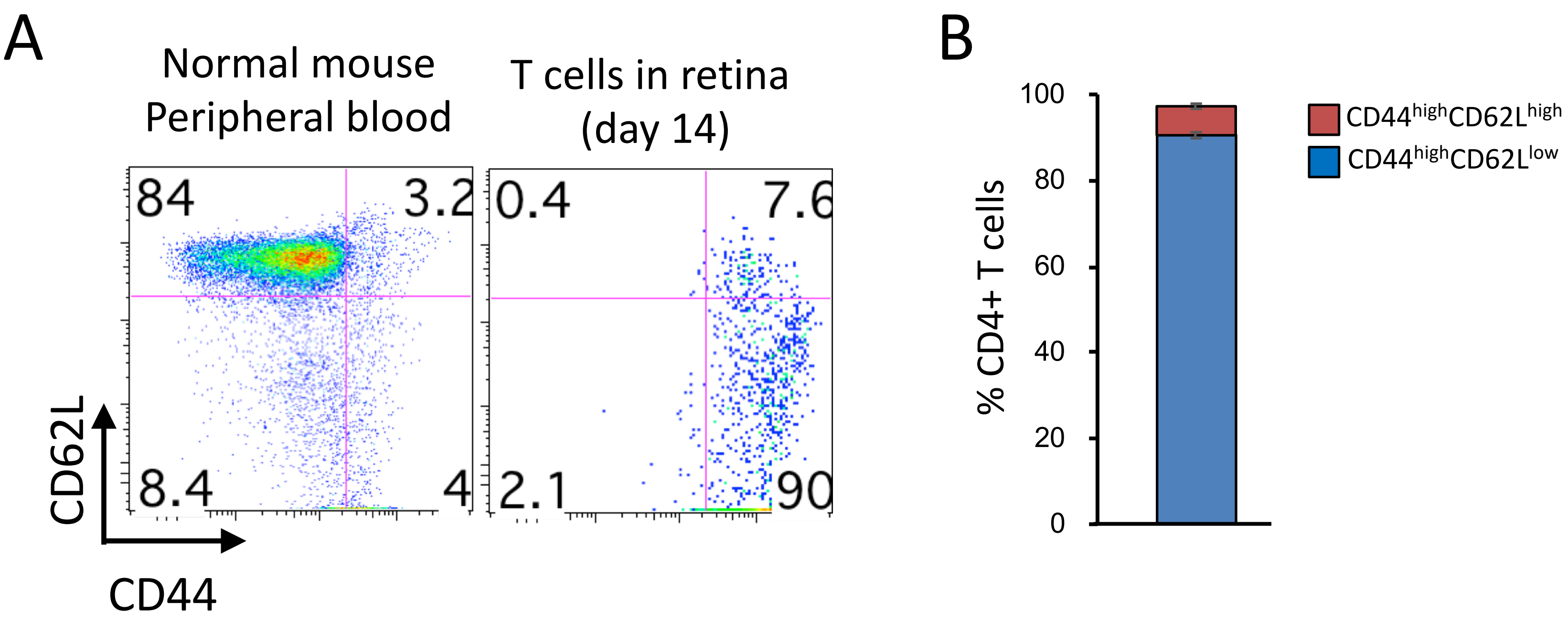

Supplementary Figure S3. CD44 and CD62L levels in retina-infiltrated CD4+ T cells

A. CD44 and CD62L levels in T cells under the mesopic condition were examined on day 14 post immunization. Peripheral blood T cells from normal mice were used for staining control of naïve T cells. FACS plots were gated on CD45+CD90.2+CD4+ cells.

B. The percentage of activated CD4 T cells within total CD4+ T cells (CD45+CD90.2+CD4+) in the retina is shown. Data represent the mean + SEM (n = 12).
